# Interference with pancreatic sympathetic signaling halts the onset of diabetes in mice

**DOI:** 10.1126/sciadv.abb2878

**Published:** 2020-08-26

**Authors:** Gustaf Christoffersson, Sowbarnika S. Ratliff, Matthias G. von Herrath

**Affiliations:** 1 La Jolla Institute for Immunology, La Jolla, CA 92037, USA.; 2 Department of Medical Cell Biology, Uppsala University, Uppsala 75237, Sweden.; 3 Science for Life Laboratory, Uppsala University, Uppsala 75237, Sweden.; 4 Novo Nordisk Research Center, Seattle, WA 98109, USA.

## Abstract

The notably lobular distribution of immune lesions in type 1 diabetes (T1D) has been hypothesized to be the result of innervation within the pancreas. To investigate whether neuroimmune interactions could explain this phenomenon, we explored the impact of sympathetic signaling in the RIP-LCMV-GP mouse model of autoimmune diabetes. In this model, the CD8^+^ T cell attack on β cells replicates a key pathogenic feature of human T1D. We found that inhibition of α_1_ adrenoceptors, ablation of sympathetic nerves, and surgical denervation all had a protective effect in this model, without affecting the systemic presence of β cell–reactive CD8^+^ T cells. In vivo multiphoton imaging revealed a local effect within pancreatic islets including limited infiltration of both macrophages and β cell–specific CD8^+^ T cells. Islet-resident macrophages expressed adrenoceptors and were responsive to catecholamines. Islet macrophages may therefore constitute a pivotal neuroimmune signaling relay and could be a target for future interventions in T1D.

## Teaser

Nerve signaling in the pancreas affects the local immune response in type 1 diabetes and can be targeted to halt disease.

## INTRODUCTION

Several studies have suggested a role for the innervation of pancreatic islets in the development of type 1 diabetes (T1D), but no clear causal relationship in human T1D has been determined. The “patchy” and lobular pattern of islet immune infiltration and β cell destruction in human pancreata (often described as alopecia- or vitiligo-like) has led to the hypothesis that specific nerves are involved in controlling the autoimmune attack (*1*). Studies have shown that pancreatic sensory neurons regulate islet inflammation in the nonobese diabetic (NOD) mouse through the release of transient receptor potential cation channel subfamily V member 1 (TRPV1) (*2*), and in streptozotocin-treated mice, pharmacological inhibition of adrenergic α_1_ receptors prevents hyperglycemia (*3*). Specific immune targeting of intra- and peri-islet nerve structures has also been found at an early stage of islet immune infiltration in mice (*4*, *5*). Within the pancreas in human T1D, there is a loss of sympathetic neurons from the islets, not only following destruction of the β cells but also at an early stage of the disease (*6*).

Most cell types from both the adaptive and innate immune systems express adrenergic receptors and can therefore receive catecholaminergic signals from sympathetic nerves (*7*). Macrophages express both α and β adrenoceptors. Ligation of α adrenoceptors tends to promote a proinflammatory phenotype (*8*), whereas ligation of β_2_ adrenoceptors has the opposite effect (*9*). T cells primarily express β_2_ adrenoceptors, and exposure to catecholamines generally increases CD4^+^ T cell activity (*10*) and limits CD8^+^ T cell activity (*11*), although discrepancies can be found in the literature depending on the context and the type of challenge.

In recent years, the field of neuroimmune research has grown, with increasing insights into the connections between the sympathetic nervous system and immune cell activity (*12*). Neuroimmune connections have been found to affect homeostatic processes in both the healthy state, such as in intestinal barrier function (*13*) and during lipolysis in adipose tissue (*14*), as well as in specific diseases where an effect on macrophages has been shown in both multiple sclerosis (*15*) and rheumatoid arthritis (*16*).

To investigate whether neuroimmune connections were also involved in the development of T1D, we investigated the influence of sympathetic nerve activity in the pancreas on the development of T1D in the rat insulin promoter–lymphocytic choriomeningitis virus–glycoprotein (RIP-LCMV-GP) mouse model of T1D. In this model, a strong viral induction of CD8^+^ T cells targeting a transgenically expressed viral antigen on the β cells results in autoreactivity and hyperglycemia within 14 days. Regimes that have been found to successfully prevent T1D in the NOD mouse are generally unsuccessful in this stringent model due to the strong antigenic reactivity toward β cells. However, in these studies, we found a notable effect both of adrenoceptor antagonists and of denervation in limiting islet inflammation and preventing hyperglycemia. Intravital imaging revealed an effect on the pancreatic macrophages, which, in turn, were found to be particularly prone to stimulation by catecholamines and could therefore regulate the local immune response.

## RESULTS

### Interfering with adrenergic signaling halts diabetes onset in RIP-LCMV-GP mice

**Fig. 1:**
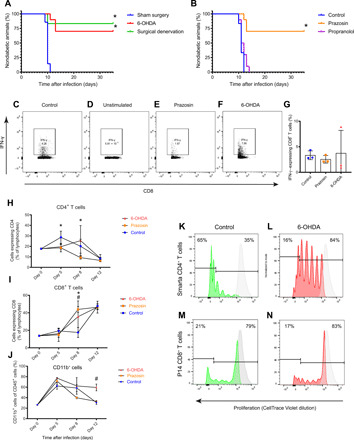
Interference with adrenergic signaling halts T1D in the RIP-LCMV-GP mouse. (**A**) Denervation of the pancreas either surgically (green line) or chemically (by 6-OHDA; red line) protected from diabetes onset in the aggressive RIP-LCMV-GP model where all animals in the sham surgery group (blue line) reached hyperglycemia within 14 days [8 to 10 mice per group, log-rank (Mantel-Cox) test]. (**B**) Antagonists to adrenoceptors were administered daily to mice following infection with LCMV. α_1_ inhibition by prazosin (orange line) was protective, whereas β inhibition by propranolol (magenta line) had no beneficial effect on the disease course compared with vehicle (blue line) control [810 mice per group, log-rank (Mantel-Cox) test]. *P < 0.05. (**C** to **G**) Treatment with prazosin or 6-OHDA did not affect the CD8^+^ T cell response to the driver antigen in this model as judged by the IFN-γ response to in vitro stimulation with GP_33–41_ in lymphocytes isolated from blood [one-way analysis of variance (ANOVA), *P < 0.05 for control versus prazosin and #*P* < 0.05 for control versus 6-OHDA]. Treatment with prazosin or 6-OHDA had significant effects on some days in the disease course (four mice per group, one-way ANOVA) on counts of circulating (**H**) CD4^+^ T cells, (**I**) CD8^+^ T cells, or (**J**) CD11b^+^ myeloid cells. CellTrace Violet–labeled GP-specific T cells were transferred on day 7 after LCMV infection, and their proliferation in the pancreas was measured on day 12. A strong effect on Smarta CD4^+^ T cells was found compared with control (**K**) following treatment with 6-OHDA (**L**), but not on P14 CD8^+^ T cells compared to control (**M**) following treatment with 6-OHDA (**N**). Representative flow histograms from groups of four mice (Mann-Whitney *U* test).

The objective of these experiments was to test the impact of sympathetic innervation of the pancreas on β cell loss and the progression of hyperglycemia in the RIP-LCMV-GP mouse. In the first set of experiments, two strategies for eliminating sympathetic innervation were tested: physical denervation—achieved by cutting an efferent tyrosine hydroxylase–positive nerve bundle innervating the pancreas at the superior mesenteric artery (fig. S1, A and B), and chemical denervation using 6-hydroxydopamine (6-OHDA), a neurotoxin that depletes peripheral sympathetic neurons. Surgical denervation was performed 10 days before infection with LCMV, and treatment with 6-OHDA was started the day after infection and repeated every third day thereafter. Surgical denervation did not affect glucose homeostasis (fig. S2A). Both modes of denervation were protective in this model. Only 15% (surgical denervation) and 27% (chemical denervation) of the animals progressed to diabetes compared with the 100% incidence in mice that underwent sham surgery or vehicle treatment (Fig. 1A).

The unexpected impact of these treatments on diabetes onset in this model prompted us to explore whether pharmacological interference with adrenergic signaling could have similar effects. We used the selective adrenoceptor α_1_ antagonist prazosin and the nonselective adrenoceptor β antagonist propranolol. These drugs were administered intraperitoneally once daily starting the day after infection with LCMV. A similar level of protection from diabetes was observed for α receptor inhibition with prazosin. However, with the β receptor antagonist propranolol, no protection was seen; instead, the animals progressed to very high blood glucose values earlier than the vehicle-treated controls (Fig. 1B and fig. S2B). This difference was not statistically significant but could point to immunosuppressive effects of signaling through β adrenoceptors.

To investigate whether the effect on diabetes incidence was due to alterations in the clearance of the LCMV virus, we isolated blood lymphocytes and assessed the interferon-γ (IFN-γ) response from CD8^+^ T cells stimulated with GP_33–41_. The IFN-γ response was similar across the groups (Fig. 1, C to G), indicating that a robust antiviral response was present in all groups and that diabetogenic T cells were present in sufficient amounts to induce disease (*17*). We also performed flow cytometry on blood following infection to assess blood leukocyte subsets to see whether any patterns could be found to explain an altered response to diabetes induction. We found significant differences in numbers of CD4^+^ T cells in the prazosin-treated group on days 5 and 8 and an increase in numbers of circulating CD8^+^ T cells in prazosin- and 6-OHDA–treated mice on day 8 (Fig. 1, H and I). Numbers of CD11b^+^ cells (CD4^−^/CD8^−^/CD19^−^/CD45^+^/CD11b^+^ of myeloid origin) were increased in blood of prazosin-treated mice on day 12 (Fig. 1J). However, we could not discern any distinct patterns to explain the local effects in the pancreas.

The local proliferative capacities of GP-specific (β cell specific) T cell receptor (TCR) transgenic CD4^+^ (Smarta; Fig. 1, K and L) and CD8^+^ (P14; Fig. 1, M and N) T cells were investigated by adoptive transfer of CellTrace Violet–labeled cells to mice undergoing diabetes induction treated with 6-OHDA or vehicle control on day 7 after infection. On day 12, pancreata were harvested, and dilution of the dye was analyzed by flow cytometry. While little effect was seen on the proliferation of β cell–specific CD8^+^ T cells, treatment with 6-OHDA significantly reduced the proliferation of CD4^+^ T cells in the pancreas.

### Intravital imaging of the pancreas reveals local effects of interfering with adrenergic signaling

**Fig. 2:**
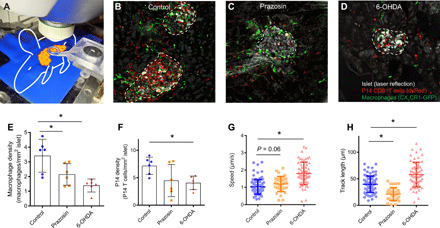
Local effects of interference with adrenergic signaling in the pancreas revealed by intravital imaging. (**A**) Intravital imaging of the mouse pancreas in anesthetized mice was performed in a confocal/multiphoton microscope using a 3D-printed vacuum window (photo credit: G. Christoffersson, Department of Medical Cell Biology, Uppsala University). (**B**) On days 10 to 12 following LCMV infection, control mice had large infiltrations in islets (gray, laser reflection) by macrophages (green, CX_3_CR1-GFP) and P14 CD8^+^ T cells (red, DsRed). Treatment with prazosin (**C**) or 6-OHDA (**D**) altered the inflammatory environment at the islet with fewer macrophages and in 6-OHDA–treated mice with fewer CD8^+^ T cells. (**E**) Macrophage numbers were significantly decreased in mice treated with 6-OHDA and prazosin (*n* = 6 mice per group, one-way ANOVA with Tukey’s multiple comparisons test). (**F**) Significant differences were seen in numbers of CD8^+^ T cells in the 6-OHDA–treated group (*n* = 6 mice per group, one-way ANOVA with Tukey’s multiple comparisons test). The behavior of the islet-specific P14 CD8^+^ T cells was altered in mice treated with prazosin and 6-OHDA with respect to their speed (**G**) and distance traveled (**H**) (values displayed are from one islet, representative of at least five mice per group, one-way ANOVA with Tukey’s multiple comparisons test). *P < 0.05.

Since blood leukocyte counts gave little insight into the mechanism of the protection from diabetes seen during total sympathetic blockade or adrenoceptor α_1_ inhibition, we turned to high-resolution intravital microscopy to study the local immune events within the islets. Using an in vivo imaging setup where the pancreas was immobilized to a coverglass by a vacuum device, long-term confocal imaging of anesthetized mice could be achieved (Fig. 2A) (*18*, *19*).

Imaging was focused on the effector cells in this model—GP-specific P14 CD8^+^ T cells (DsRed), antigen-presenting cells (APCs), macrophages, and dendritic cells (CX_3_CR1-GFP). Imaging was performed on days 10 to 12 following virus infection, and control mice showed a response similar to what is normally observed in this model at this time point: a high activity and large infiltration of CD8^+^ T cells as well as a large infiltration of CX_3_CR1^+^ macrophages and dendritic cells (Fig. 2, B, E, and F, and movie S1) (*19*). In pancreata of prazosin-treated mice, a reduced number of APCs was observed along with a tendency toward fewer P14 CD8^+^ T cells at the islets (Fig. 2, C, E, and F). In the 6-OHDA–treated mice, there was a significant reduction in both APCs and P14 CD8^+^ T cells at the islets (Fig. 2, D, E, and F).

**Fig. 3:**
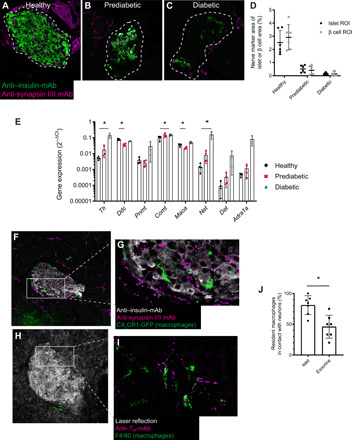
Islet nerves are in close juxtaposition to macrophages and are lost during onset of T1D. During onset of T1D in RIP-LCMV-GP mice, nerves in the (**A**) healthy islets (uninfected) were gradually lost in (**B**) prediabetic (day 7 after infection) and (**C**) diabetic (day 14 after infection) animals [anti-insulin (green) and anti-synapsin (magenta)]. mAb, monoclonal antibody. An islet region of interest (ROI) was defined [dashed line in (A) to (C)], and a β cell ROI was defined by the anti-insulin signal (green). (**D**) Intra-islet nerves were lost uniformly within the islet and not limited to nerves close to β cells. (**E**) The expressions of genes related to the sympathetic nerve system were altered in the pancreas during onset of T1D. (**F** and **G**) Immune fluorescence imaging of pancreas sections from uninfected mice revealed that islet-resident macrophages (green, CX_3_CR1-GFP) were in close juxtaposition to intra-islet nerves using the general nerve marker synapsin (magenta, anti–synapsin-mAb). When staining for sympathetic nerves using anti–tyrosine hydroxylase mAb (magenta) (**H** and **I**), this was also found to be true for tyrosine hydroxylase–positive nerves. (**J**) Islet-resident macrophages were more frequently in close contact with nerves than macrophages residing in the exocrine pancreas (*n* = 7 pancreata, two-tailed Wilcoxon’s nonparametric test).

The local behavior of immune cells gives further information regarding their level of activation and their propensity to interact with, for example, target tissue (i.e., β cells) and APCs. The behavior of the P14 CD8^+^ T cells in the two treatment groups was different from that in the controls. In mice receiving 6-OHDA, T cells migrated with greater speeds (Fig. 2G) and over longer distances (Fig. 3H and movie S2). In other studies, this has led to less interaction with target cells and APCs (*19*), which is in line with the notion that β cells are spared in these mice. P14 CD8^+^ T cells in mice treated with prazosin were, on the other hand, less motile with lower speed (Fig. 2G) and less distance covered during migration (Fig. 2H and movie S3). How this correlates to the decreased level of β cell destruction is less clear, but could point to a lower degree of activation in these cells.

### Islet-resident macrophages are in close contact with nerves, and nerves are lost during onset of T1D

Similar to what has been previously reported (*5*), we found that islet nerves disappeared at an early stage of T1D progression in the RIP-LCMV-GP model. Pancreatic islets in mice are usually densely innervated (Fig. 3A), but by day 7 after infection, when the first signs of β cell loss could be observed (“prediabetic”; blood glucose values, ~180 to 250 mg/dl), most intra-islet nerves were no longer present (Fig. 3B). At 14 days after infection, when the mice were overtly diabetic (blood glucose values, >500 mg/dl), hardly any nerve structures were present in the islets (Fig. 3C). The loss of nerves was not limited to structures associated with β cells but occurred throughout the islet (Fig. 3D).

To get a sense of the activity of the sympathetic nervous system during the onset of T1D in the RIP-LCMV-GP mouse model, a range of genes related to adrenergic signaling were assessed at three different time points used in this study (healthy, no infection; prediabetic, 7 days after infection; and diabetic, 14 days after infection). Despite the loss of intra-islet nerves, a trend for an increase in expression levels of most of the assessed genes was found during the disease course and was statistically significant for *Th* (tyrosine hydroxylase), *Comt* (catechol-*O*-methyltransferase), *Net* (norepinephrine transporter), and *Adra1a* (adrenoceptor 1α) (Fig. 3E).

When performing coimmunofluorescence staining for neural markers and immune cells in pancreatic islets, it was evident that a general neural marker was expressed close to intra-islet macrophages in healthy, uninfected mice (Fig. 3, F and G). The same pattern was evident also for sympathetic neurons in tissue sections stained with anti–tyrosine hydroxylase (Fig. 3, H and I). Even though this juxtaposition is, to some extent, to be expected due to the fact that both macrophages and nerves are often situated close to vasculature, the extent of juxtaposition was much greater in islets compared with exocrine pancreatic tissue (Fig. 3J). Because the influence of neuronal signaling on immune cells in general and macrophages in particular is becoming more and more evident (*20*), we decided to investigate the influence of sympathetic signaling on islet macrophages.

### Macrophages are required for the onset of T1D and respond to catecholamines

**Fig. 4:**
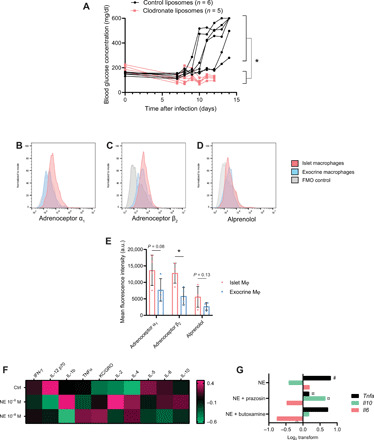
The islet macrophage as a putative regulator of sympathetic input. (**A**) Depletion of macrophages from RIP-LCMV-GP mice by clodronate liposomes during onset of T1D was protective against the development of hyperglycemia (Student’s *t* test on day 12, *P < 0.05 on day 12). (**B** to **E**) Pancreatic macrophages in healthy, uninfected mice expressed both α_1_ and β_2_ adrenoceptors, and intra-islet macrophages had a higher expression of β_2_ adrenoceptors (C) and tendencies of higher expression of α_1_ adrenoceptors (B) than exocrine macrophages (flow plots representative of *n* = 4 mice). (D) A biotinylated nonselective β blocker, alprenolol, was used as an additional detection method for β adrenoceptors. Mean fluorescence intensities of receptor staining from four mice (Student’s *t* test, *P < 0.05) are presented in (E). FMO, fluorescence minus one. a.u., arbitrary units. (**F**) Isolated islet macrophages were stimulated with 10^−6^ and 10^−8^ M norepinephrine (NE) in vitro and shifted their expression of cytokines. The heatmap displays log_2_ transforms of quantitative cytokine concentrations in cell lysates (absolute values in fig. S3). (**G**) Islet-resident macrophages were isolated and stimulated with 10^−8^ M NE in the presence or absence of an α_1_ (prazosin) or a β_2_ (butoxamine) adrenoceptor antagonist (*n* = 3 mice in two independent experiments, one-way ANOVA). #*P* < 0.05 compared to unstimulated control and ¤*P* < 0.05 compared to the NE-stimulated group.

Studies have found that macrophages are crucial for the development of diabetes in both the NOD mouse (*21*, *22*) and the RIP-LCMV-GP mouse (*23*). We also found that when we depleted macrophages using clodronate liposomes in the RIP-LCMV-GP model, the mice were protected from the development of diabetes (Fig. 4A). The viral induction of autoimmunity faced a challenge when macrophages were depleted, which was possibly due to the lack of viral clearance when a large portion of the APCs were lost. Clodronate-treated mice had to be euthanized on day 12 after infection to satisfy humane end points, but no animals in this group had developed hyperglycemia at this point.

Intra-islet macrophages in mice have been found to exhibit a classically activated phenotype in healthy mice, whereas resident macrophages in the exocrine pancreas are more heterogeneous and are skewed toward an alternatively activated phenotype (*24*). Here, we found a further dichotomy: In our experiments, intra-islet macrophages expressed adrenoceptors α_1_ and β_2_ at higher levels than exocrine macrophages (Fig. 4, B to E).

Islet macrophages were isolated from islets and exocrine tissue, respectively. These macrophages were immediately cultured for 4 hours in the absence or presence of high (1 × 10^−6^ M) or low (1 × 10^−8^ M) concentrations of norepinephrine, and cell lysates were analyzed for inflammation-related cytokines using a quantitative multiplexed assay. In this isolated system, treatment with norepinephrine had robust effects on the expression of a range of cytokines (Fig. 4D and fig. S3). The high concentration of norepinephrine can be considered to be the concentration experienced close to a nerve terminal and has, in other studies, been observed to have anti-inflammatory effects on immune cells due to the primary activation of β adrenoceptor signaling (*25*). The lower concentration has been observed to have the opposite effect due to the primary activation of α adrenoceptor signaling. The high concentration of norepinephrine tends to reduce T helper 1 (T_H_1) cytokine expression and increase the expression of T_H_2 cytokines. The opposite occurs at the low concentration of norepinephrine. The effect of adrenoceptor inhibition was tested in a similar setup where islet-resident macrophages were stimulated with norepinephrine in the presence or absence of prazosin (α_1_ adrenoceptor antagonist) or butoxamine (β_2_ adrenoceptor antagonist). Blocking α_1_ receptors resulted in increased expression of the *Tnfa* gene and increased expression of the *Il10* gene, whereas blocking β_2_ receptors sustained a high expression of *Tnfa* and did not affect *Il10* expression (Fig. 4E). These data therefore point to the possibility that there is a central contribution to T1D development by macrophages and a possible immune-regulatory influence from sympathetic nerves, which is relayed through islet-resident macrophages.

## DISCUSSION

The etiology of T1D is still a matter of intense research. Neither the triggers for the initiation of the disease nor a complete understanding of the natural course of the disease is yet known to the research community. The nervous system has been suggested as the reason why the different lobules of the pancreas seem to be affected in distinct ways by immune destruction (*1*). In this study, we investigated how interference with sympathetic signaling affects the immune response toward β cells in the RIP-LCMV-GP mouse model of T1D. We found that both sympathetic denervation and adrenoceptor α_1_ inhibition halted the autoaggressive immune response in the pancreas and that the majority of treated mice in these conditions remained normoglycemic. It was notable that these interventions could almost completely disrupt disease development in a model where β cell destruction is driven by highly activated antiviral CD8^+^ T cells and thus historically has been difficult to influence (*26*). Our further investigations indicated that the potential effect of macrophages as a cellular neuroimmune relay within the pancreatic islets is dominant in the early stages of disease development.

A growing body of work in the relatively new field of neuroimmunity has revealed that nervous signaling affects immune cell behavior at peripheral sites (*12*). This has been shown both during steady-state homeostatic events and during disease (*20*). One of the most described examples of neuroimmune communication is via the vagus nerve, where early experiments using electrical vagus nerve stimulation have demonstrated that acetylcholine suppresses the release of inflammatory cytokines from macrophages and dampens an endotoxin-induced systemic inflammatory response (*27*). More recent studies have shown homeostatic effects of sympathetic innervation of the gut where intestinal macrophages control neuron activity and peristalsis (*28*) and response to bacterial infections (*13*).

Studies investigating the potential of intervening with neuronal input have used electrostimulation of the vagus nerve to reduce inflammation in patients with rheumatoid arthritis (*29*) and to induce remission in patients with Crohn’s disease (*30*). Vagal stimulation may have off-target effects due to the vast afferent and efferent reach of this nerve and might therefore not be suitable for T1D treatment. A recent study in mice took a different approach where sympathetic nerves projecting into lymph nodes draining the pancreas were targeted with electrostimulation and were able to show decreased incidence of T1D in the NOD mouse (*31*). Targeting local organ innervation may be a way to take the field further, especially because recent advances in materials technology could potentially allow for the development of miniaturized implantable electrostimulators (*31*).

The influence of pancreatic innervation on T1D pathogenesis has not yet been evaluated to any great extent. Intra-islet TRPV1^+^ sensory neurons have been shown to affect islet inflammation in the NOD mouse (*2*), and these neurons were also found to be targets for the autoimmune response alongside β cells (*4*). Sympathetic nerves have been found to be absent from islets of donated pancreata from patients with T1D (*6*). Whether this is a consequence of the immune targeting of the β cells or the result of events earlier in the development of the disease is however not clear. To our knowledge, the only previous example of efforts to interfere pharmacologically with sympathetic signaling in models of T1D was the targeting of the α_1_ receptor in multiple low-dose streptozotocin-treated mice (*3*). In that study, prazosin was found to protect from hyperglycemia by limiting immune infiltration through effects on vasculature and microedema formation in islets.

It was recently described that the pancreatic islets of mice contain a fairly unique subset of resident macrophages during steady state, which are in a proinflammatory state (as opposed to the resident macrophages in the acinar exocrine tissue in the pancreas, which are more skewed toward an anti-inflammatory phenotype) (*24*, *32*). Macrophages have also been found to have a pivotal role in the initiation of the immune events, leading to the development of diabetes and hyperglycemia. For example, the increased expression of neuron-associated genes seen during disease onset in our model despite the gradual loss of nerves might be a consequence of the increased activity and influx of immune cells expressing these factors. Depletion of macrophages either in the NOD mouse (*21*, *22*) or in the RIP-LCMV-GP mouse (*23*) significantly lowers the incidence of diabetes. We depleted macrophages in the RIP-LCMV-GP model and found a strong tendency toward protection from diabetes, which supports previously published findings. These experiments had to be aborted prematurely due to reaching a humane end point, probably due to the fact that macrophages would normally be involved in the antiviral clearance of LCMV and that the mice were therefore suffering from a high viral load. Overall, macrophages seem to have a crucial role in the initiation of immune events in T1D.

In the current study, pharmacological inhibitors of α and β receptors had opposing effects on diabetes incidence. Treatment with prazosin provided protection from the development of T1D, whereas treatment with propranolol exacerbated disease progression and led to very high blood glucose values. These divergent effects of catecholaminergic signaling of α and β receptors in immune cells have been described in multiple systems. Generally, α receptor ligation leads to an inflammatory response, and β receptor ligation results in a more anti-inflammatory response (*25*). In situations close to a source of norepinephrine (e.g., a sympathetic nerve terminal) where the concentration is high, β adrenoceptor signaling is dominant, whereas further away, α adrenoceptor signaling increases in influence. We observed events that, at first, seem counterintuitive, i.e., protection by α_1_ adrenoceptor antagonization and denervation, but not by β adrenoceptor antagonization. The protective effect of denervation and, thus, the limitation of sympathetic signaling at the islets may point to a tonic inhibitory effect on islet macrophages caused by catecholaminergic signals. Immune-dampening effects by denervation have been observed in other inflammatory diseases and models. In humans affected by unilateral stroke, it has been observed that only neurologically intact limbs are affected by subsequent rheumatoid arthritis (*33*). This has also been confirmed by denervation in mouse models (*34*).

To conclude, we have found evidence for the influence of sympathetic nerve signaling on the development of T1D in a mouse model. We observed local immune regulatory effects from denervation and α_1_ adrenoceptor antagonism, which may lead to novel treatment strategies for T1D, including the possibility of local electrostimulation. The development of more refined experimental models will be required to investigate the role of the islet macrophage in a neuroimmune relay and as a key player in T1D pathophysiology.

## MATERIALS AND METHODS

### Animals

C57Bl/6J mice were purchased from the Jackson laboratories. All other strains were bred in-house under specific pathogen–free conditions at the La Jolla Institute for Allergy and Immunology, on C57Bl/6J background. Transgenic RIP-LCMV-GP mice have been described previously (*35*). TCR transgenic strains P14 (*36*) and Smarta mice were bred on DsRed or Ly5.1 (B6.SJL-*Ptprc^a^ Pepc^b^*/BoyJ)–expressing backgrounds. B6.129P-*Cx3cr1^tm1Litt^*/J mice (CX_3_CR1^GFP/GFP^) were bred with RIP-LCMV-GP mice. Female and male mice were used at 8 to 16 weeks of age. Mice were housed in biosafety level 2 facilities after LCMV infection. All animal experiments were approved by the Institutional Animal Care and Use Committee at the La Jolla Institute for Allergy and Immunology or the Swedish Laboratory Animal Ethical Committee, Uppsala (permit no. 5.9.18-03603/2018).

### Virus

The Armstrong strain of LCMV was used. Virus was grown, purified, and titrated as described previously (*37*).

### Treatment

Treatment was started on the day after LCMV infection. Prazosin [1 mg/kg body weight (bw), dissolved in ddH_2_O; Sigma-Aldrich) and propranolol (2 mg/kg bw, dissolved in phosphate-buffered saline; Sigma-Aldrich) were administered intraperitoneally daily for 10 days. 6-OHDA hydrobromide (100 mg/kg bw, dissolved in 0.1% ascorbic acid solution; Sigma-Aldrich) was administered intraperitoneally every third day for 10 days (a total of four injections).

### Pancreatic denervation

Mice were anesthetized by spontaneous inhalation of isoflurane and were given analgesia preoperatively and postoperatively according to protocol (buprenorphine, 0.1 mg/kg bw). An incision in the right flank was made and held up by wound retractors. Sterile cotton swabs were used to move intestines to access the inferior pancreaticoduodenal artery. A T_H_^+^ nerve structure along this vessel was exposed, and a 2- to 3-mm section was cut and removed (see fig. S1 for images showing the anatomical location and T_H_ staining). Following suturing, mice were allowed to heal for 10 days before LCMV infection. Denervation was confirmed by significant reduction in T_H_^+^ nerve structures in the pancreas.

### Adoptive cell transfers

Single-cell suspensions were prepared from the spleen and lymph nodes. Red blood cell lysis was performed, and CD8^+^ T cells were isolated using negative selection (Miltenyi Biotec). Purity was confirmed by flow cytometry and was consistently about 95% TCR^+^CD8^+^ of total cells. Cells (1 × 10^6^) were then transferred via retroorbital injection to mice 1 day before infection.

For the in vivo T cell proliferation assay, isolated cells were transferred in single-cell suspensions into mice through retroorbital injections. Purified P14 CD45.1^+^ CD8^+^ T cells and Smarta CD45.1^+^ CD4^+^ T cells (isolated by negative selection; Miltenyi Biotec) were stained in vitro using the intracellular dye CellTrace Violet (Thermo Fisher Scientific) according to the manufacturer’s instructions. Cells (1 × 10^6^) were then transferred into mice undergoing the viral diabetes induction protocol on day 7. On day 12, pancreas and pancreatic draining lymph nodes were harvested, digested, and subjected to flow cytometry to examine dye dilution.

### Induction of autoimmune diabetes

Diabetes was induced in transgenic animals by infection with 2 × 10^5^ plaque-forming units of LCMV-Arm intraperitoneally. Blood glucose levels were measured on regular intervals up to day 21 using test strips (OneTouch Ultra, LifeScan). Mice with blood glucose levels above 250 mg/dl on two consecutive measurements were considered diabetic.

### Intravital imaging

A previously described protocol for confocal and multiphoton microscopy of the mouse pancreas (*38*, *39*) was adapted to incorporate the use of a three-dimensionally (3D) printed custom-made immobilization device using a vacuum [Fig. 2A, described in (*18*)]. Briefly, mice were anesthetized with an initial dose of ketamine hydrochloride (90 mg/kg) and xylazine hydrochloride (15 mg/kg), which was iterated as needed during the course of the experiment to ensure surgical anesthesia was maintained. A longitudinal incision was made in the left flank exposing the tail of the pancreas. The vacuum device was then lowered in place to immobilize the pancreas to a coverslip for imaging using a Leica HC Fluotar L 25×/0.95 W objective on a Leica SP8 confocal microscope with a spectral detector setup with four visible laser lines. The pancreatic islets were in most experiments visualized using the reflected light from the 633-nm laser line. The granularity of the endocrine cells gives them a reflective signature dissimilar to one of the exocrine pancreas, eliminating the need for genetic or antibody labeling.

### Intracellular cytokine staining of blood lymphocytes

Blood samples were collected from mice infected with LCMV in the vehicle-, prazosin-, and 6-OHDA–treated groups on day 8 after infection. Red blood cells were removed by hypotonic lysis. Cells were stimulated for 1 hour with GP_33–41_ peptide (GenScript), phorbol myristate acetate (PMA)/ionomycin, or left unstimulated. Cytokine transport was inhibited by brefeldin A. Cells were then fixed, permeabilized (intracellular permeabilization kit; eBioscience), and stained for flow cytometry according to the antibody list below.

### Image analysis

Intravital recordings and micrographs were analyzed using Imaris 7.0 (Bitplane) and Fiji/ImageJ (National Institutes of Health). Migration analyses were made using the built-in tool of Imaris.

### Depletion of macrophages

Mice were infected with LCMV on day 0 and received an intraperitoneal injection of 200 μl of clodronate liposomes (FormuMax) or empty control liposomes on day 6 and an intraperitoneal injection of 100 μl of the respective treatments on day 8.

### Quantitative cytokine measurements

Cells were lysed and homogenized in radioimmunoprecipitation assay buffer. Homogenates were analyzed on a 10-plex antibody–based protein assay [V-PLEX proinflammatory panel; MesoScale Discovery (MSD)] and read on a Sector Imager 2400 (MSD). Total protein content was determined using the Bradford assay (Sigma-Aldrich).

### Quantitative polymerase chain reaction of pancreata

Pancreata from mice were harvested and processed for RNA isolation according to the kit manufacturer’s protocol (Norgen Biotek). cDNA was synthesized using the High-Capacity cDNA Reverse Transcription Kit (Thermo Fisher Scientific), and results were obtained on a QuantStudio Digital polymerase chain reaction system (Thermo Fisher Scientific). Primer sequences are available in table S1.

### Flow cytometry

All flow cytometry experiments were run on BD LSRII Fortessa (BD Biosciences) or Cytoflex S (Beckman Coulter) instruments. Antibodies used were raised against CD8a (53-6.7), CD4 (L3T4, RM4-5), CD3e (500A2), CD11b (M1/70), CD45.1 (A20), and IFN-γ (XMG1.2), all from BD Pharmingen; F4/80 (BM8), CD45.2 (104), CD19 (1D3), and major histocompatibility complex II (M5-114.15.2), all from eBioscience; and α_1_ adrenoceptor [EPR9691(B) ab137123] and β_2_ adrenoceptor [EPR707(N) ab182136], both from Abcam. A biotinylated nonselective β blocker (biotin-alprenolol; CellMosaic) was used as an alternative approach to detect β adrenoceptors. eBioscience strepatavidin phycoerythrin (Thermo Fisher Scientific) was used for fluorescence detection. Dead cells were detected using the Aqua Live/Dead Staining Kit (Life Technologies). All flow cytometry data were initially gated on lymphocytes>singlets in two steps>live cells>. Data were analyzed using FlowJo software (TreeStar).

### Pancreatic islet isolation

Mouse islets were isolated from recipients by collagenase digestion and a density gradient method described earlier (*40*). Briefly, the mice were anesthetized by spontaneous inhalation of isoflurane followed by cervical dislocation. Ice-cold collagenase solution (from *Clostridium histolyticum*, 2.5 mg/ml, Roche Diagnostics, in Hanks’ balanced salt solution) was then injected into the pancreas via the common bile duct. The pancreas was then removed and placed in a 37°C water bath for 18 min. Islets were separated from exocrine tissue by density gradient centrifugation (Histopaque-1077 and RPMI 1640; Sigma-Aldrich). Last, purified islets were handpicked and maintained free floating in islet culture medium [RPMI 1640 with added d-glucose (11.1 mmol/liter), l-glutamine (2 mmol/liter) (Sigma-Aldrich), benzyl penicillin (100 U/ml; Roche Diagnostics), streptomycin (0.1 mg/ml), and 10% (v/v) fetal calf serum (Sigma-Aldrich)].

### Isolation of pancreatic macrophages

The separated fractions of the pancreas (islets and exocrine tissue) were dissociated into single cells using a nonenzymatic approach (cell dissociation buffer; Thermo Fisher Scientific). Single-cell suspensions were then submitted to an antibody-based macrophage isolation protocol (F4/80 microbeads; Miltenyi Biotec) twice to increase purity to approximately 90%.

### In vitro stimulation of macrophages

Islet macrophages were cultured immediately following isolation at 5000 cells per well in 24-well plates. Cells were cultured in presence of norepinephrine (1 × 10^−6^ or 1 × 10^−8^ M; Sigma-Aldrich) for 4 hours before being harvested for further analysis of cytokines or mRNA (Norgen Biotek RNA isolation kit). Antagonists prazosin (Sigma-Aldrich) and butoxamine (Sigma-Aldrich) were added at concentration of 1 × 10^−8^ M.

### Statistics

Data are expressed as means ± SEM. Comparisons between the different groups were performed with two-tailed, unpaired, nonparametric, Mann-Whitney *U* tests (two groups), one-way analysis of variance (ANOVA), or log-rank tests (specified in the respective figure legends). A *P* value <0.05 was considered significant.

## Supplementary Material

abb2878_Movie_S3.avi

abb2878_SM.pdf

abb2878_Movie_S2.avi

abb2878_Movie_S1.avi

## SUPPLEMENTARY MATERIALS

Supplementary material for this article is available at http://advances.sciencemag.org/cgi/content/full/6/35/eabb2878/DC1

## Appendix

View/request a protocol for this paper from *Bio-protocol*.
