# Supplementary material for: Interference with pancreatic sympathetic signaling halts the onset of diabetes in mice
Source: Sci Adv. 2020 Aug 26;6(35):eabb2878. doi: 10.1126/sciadv.abb2878 (PMC7531904; doi:10.1126/sciadv.abb2878)
Supplement: abb2878_SM.pdf [file abb2878_SM.pdf]

Science Advances
AAAS

advances.sciencemag.org/cgi/content/full/6/35/eabb2878/DC1

# Supplementary Materials for

## Interference with pancreatic sympathetic signaling halts the onset of diabetes in mice

Gustaf Christoffersson*, Sowbarnika S. Ratliff, Matthias G. von Herrath*

*Corresponding author. Email: matthias@lji.org (M.G.v.H.);
gustaf.christoffersson@scilifelab.uu.se (G.C.)

Published 26 August 2020, *Sci. Adv.* **6**, eabb2878 (2020)
DOI: 10.1126/sciadv.abb2878

**The PDF file includes:**

Table S1
Figs. S1 to S3
Legends for movies S1 to S3

**Other Supplementary Material for this manuscript includes the following:**

(available at advances.sciencemag.org/cgi/content/full/6/35/eabb2878/DC1)

Movies S1 to S3

**Supplemental Table 1.** Primer sequences

| Gene name | Primer sequence (5'-3') |
|---|---|
| *Adra1a* | F- CTGCCATTCTTCCTCGTGAT<br>R- GCTTGGAAGACTGCCTTCTG |
| *Comt* | F- GCAGTGATTCGGGAGTACAG<br>R- TAGCGGTCTTTCCAGTGGTC |
| *Dat* | F- CGGTGGCAGCTCACAGC<br>R- TGGAGAAGGCGATCAGCAC |
| *Ddc* | F- AGGGCAGAGAAAGAATGAAAGCA<br>R- GGAGTGGTAGTTATTTTTCTCTTTCCAGTTT |
| *Gapdh* | F- GGGTCCCAGCTTAGGTTCATC<br>R- CCAATACGGCCAAATCCGTTC |
| *Il6* | F- TAGTCCTTCCTACCCCAATTTCC<br>R- TTGGTCCTTAGCCACTCCTTC |
| *Il10* | F- CAGAGCCACATGCTCCTAGA<br>R- TGTCCAGCTGGTCCTTTGTT |
| *Maoa* | F- AGTGAGCGAACGGATAATGG<br>R- TGTTCATGGTTCAGCGTCTC |
| *Net* | F- CCATACCAAATACTCCAAATACAAG<br>R- CGTGAAGAGTTTCCGGTGTCGCTT |
| *Pnmt* | F- GCCTACCTCCGCAACAACTA<br>R- GTCATGGTGATGTCCTCAAAGT |
| *Th* | F- AAGATCAAACCTACCAGCCG<br>R- TACGGGTCAAACTTCACAGAG |
| *Tnfa* | F- GAGAAAGTCAACCTCCTCTCTG<br>R- GAAGACTCCTCCCAGGTATATG |

Supplemental Figure 1

A

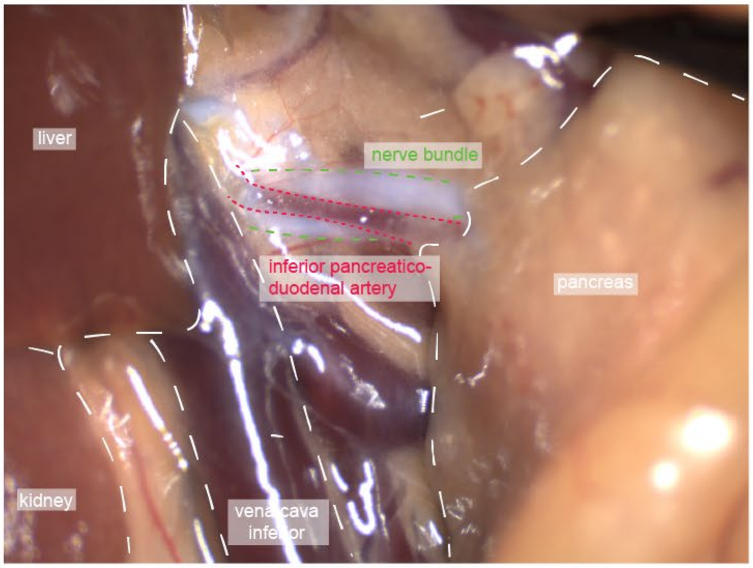


B

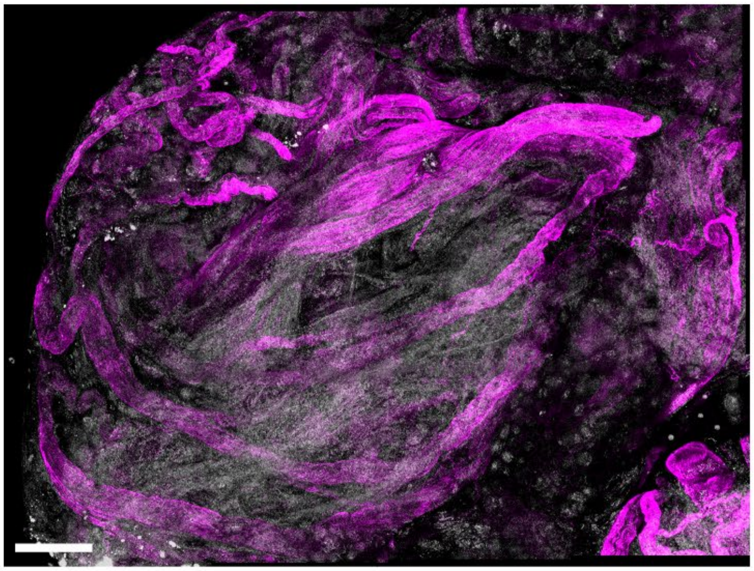

**Resection of a nerve bundle innervating the pancreas. (A)** Anatomical location of the nerve bundle entering the pancreas (photo credit: G. Christoffersson, Dept. of Medical Cell Biology, Uppsala University). **(B)** A resected section of the nerve bundle was whole mount-stained fortyrosine hydroxylase (TH) showing that these nerves were sympathetic. The image is a z-projected confocal tile scan of a part of the resected area. TH in magenta and laser reflection in gray shows connective tissue. Bar is 100 μm.

Supplemental Figure 2

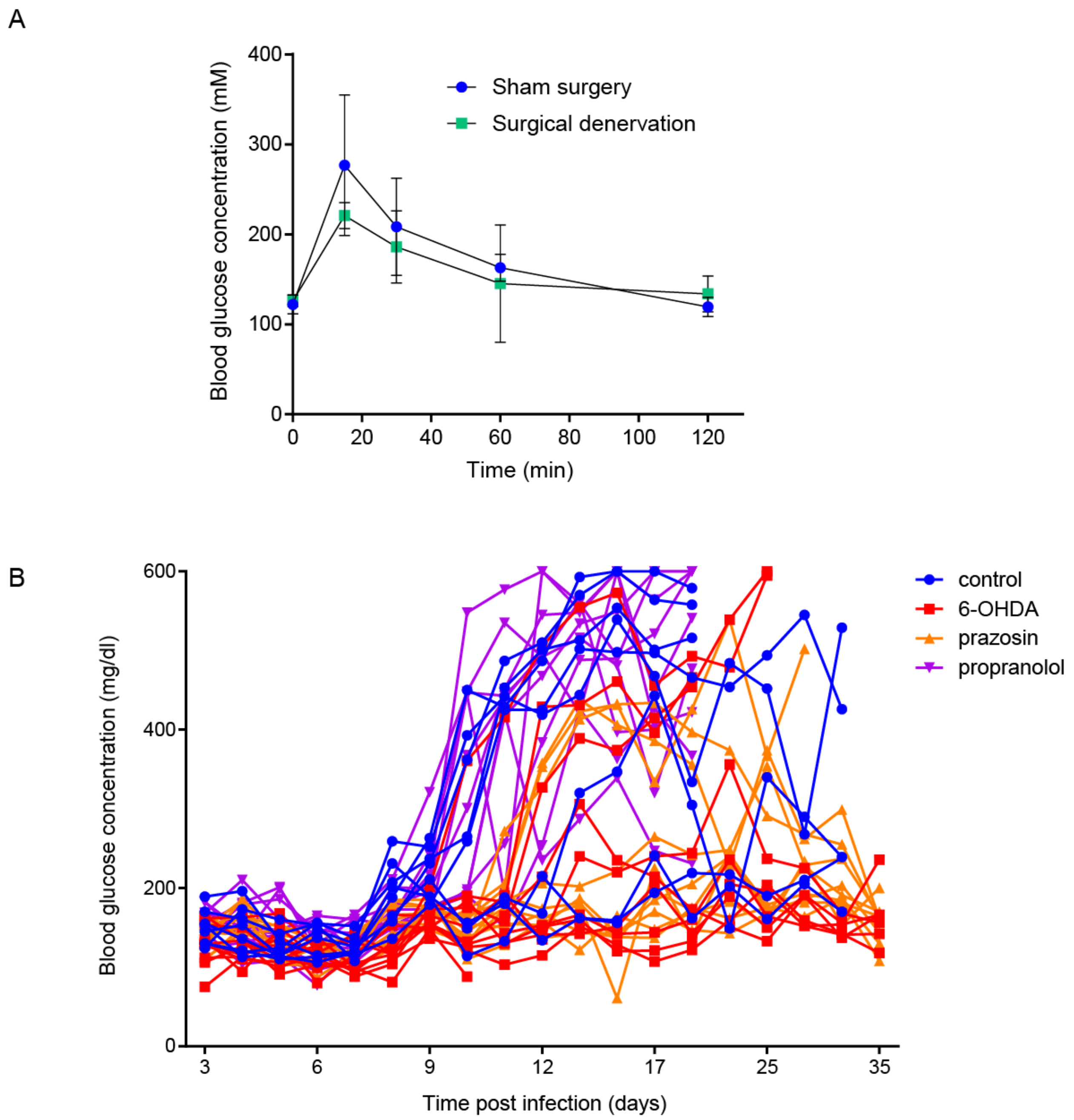


**Glucose homeostasis in inhibition experiments. (A)** No differences in glucose homeostasis in intraperitoneal glucose tolerance tests were observed following surgical denervation of the pancreas (n=4 mice per group). **(B)** Data from Fig 1 in the manuscript text shown here as individual values from one representative experiment. Note the rapid increase in blood glucose concentration in the propranolol-treated group where animals were sacrified prematurely due to reached humane endpoints.

Supplemental Figure 3

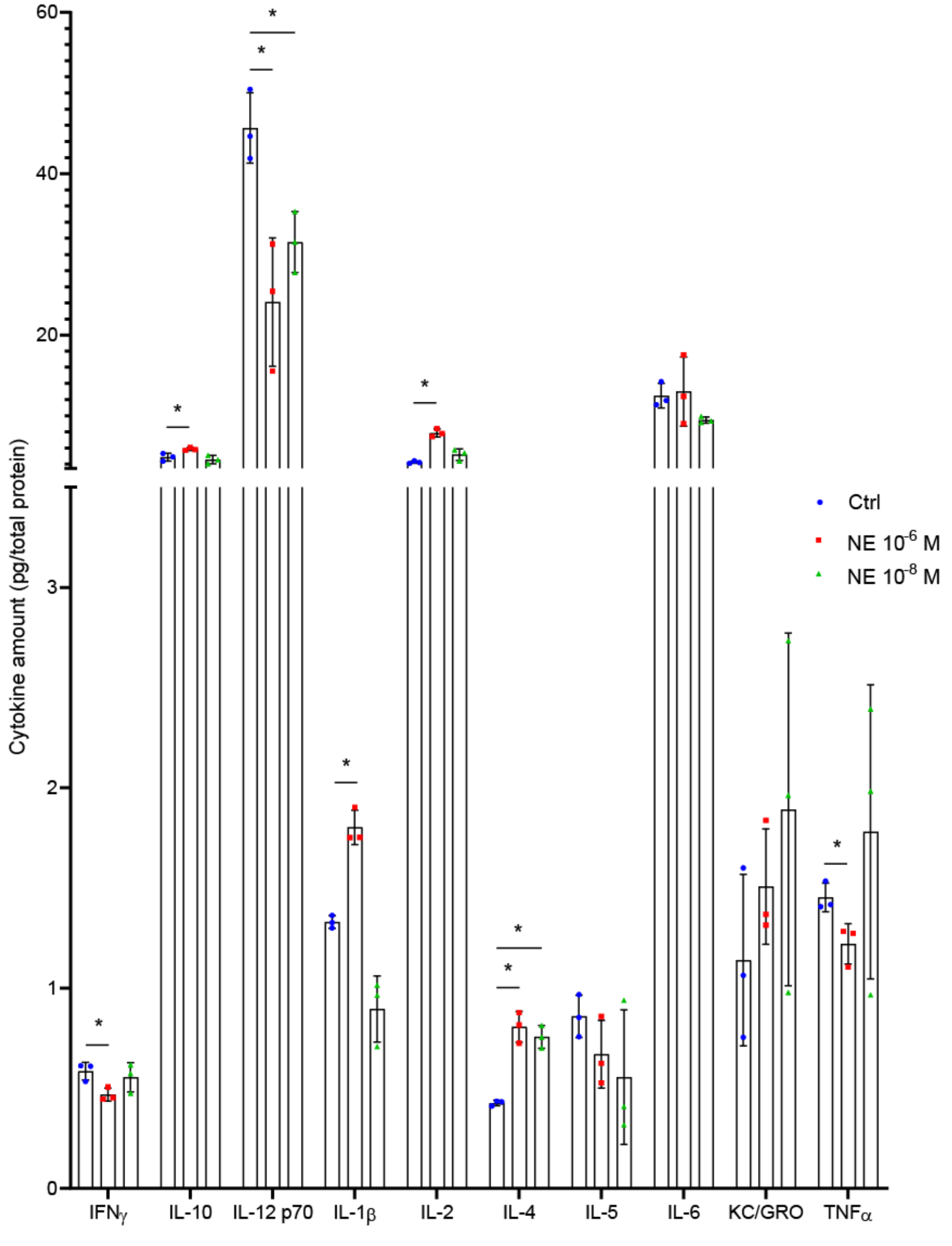


**Absolute values from quantitative cytokine measurements of stimulated islet macrophages.** Values in this graph correspond to the heatmap presented in Fig 3L. Data are from 3 individual mice and are representative of two experiments (two-way ANOVA).

## LEGENDS TO SUPPLEMENTAL VIDEOS

**Supplemental Video 1. Immune cell behavior in the pancreas of a vehicle-treated LCMV-RIP-GP mouse.** Intravital confocal video (z-projection) of two islets (blue, laser reflection), macrophages (green, $CX_3CR1$-GFP), and P14 $CD8^+$ T cells (red, dsRed). Time lapse recording, z-stacks acquired every 20 s.

**Supplemental Video 2. Immune cell behavior in the pancreas of a 6-OHDA-treated LCMV-RIP-GP mouse.** Intravital confocal video (z-projection) of two islets (blue, laser reflection), macrophages (green, $CX_3CR1$-GFP), and P14 $CD8^+$ T cells (red, dsRed). Time lapse recording, z-stacks acquired every 20 s.

**Supplemental Video 3. Immune cell behavior in the pancreas of a prazosin-treated LCMV-RIP-GP mouse.** Intravital confocal video (z-projection) of two islets (blue, laser reflection), macrophages (green, $CX_3CR1$-GFP), and P14 $CD8^+$ T cells (red, dsRed). Time lapse recording, z-stacks acquired every 20 s.
